# Predicting asymmetric phospholipid microstructures in solutions†

**DOI:** 10.1039/d0ra03732j

**Published:** 2020-06-26

**Authors:** Yue Shan, Yongyun Ji, Xianghong Wang, Linli He, Shiben Li

**Affiliations:** Department of Physics, Wenzhou University Wenzhou Zhejiang 325035 China shibenli@wzu.edu.cn

## Abstract

Asymmetric phospholipid microstructures, such as asymmetric phospholipid membranes, have potential applications in biological and medicinal processes. Here, we used the dissipative particle dynamics simulation method to predict the asymmetric phospholipid microstructures in aqueous solutions. The asymmetric phospholipid membranes, tubes and vesicles are determined and characterized by the chain density distributions and order parameters. The phase diagrams are constructed to evaluate the effects of the chain length on the asymmetric structure formations at equilibrium states, while the average radius of gyration and shape factors are calculated to analyze the asymmetric structure formations in the non-equilibrium processes. Meanwhile, we predicted the mechanical properties of the asymmetric membranes by analyzing the spatial distributions of the interface tensions and osmotic pressures in solutions.

## Abstract 2

Two types of phospholipid molecules self-assemble to form an asymmetric membrane in aqueous solution.

## Graphical abstract

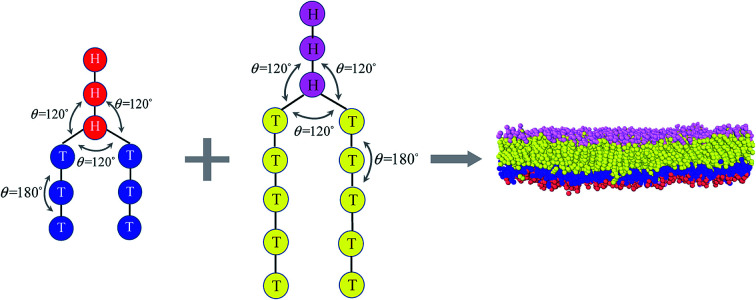

## Introduction

Asymmetric microstructures, such as asymmetric membranes, possess asymmetric structures on each side.^1^ Such asymmetric microstructures result in unusual properties, including automatic cleaning, unidirectional transport, and water–oil separation, and they differ from those in common membranes.^2–5^ For example, an asymmetric membrane can be used for unidirectional transmission due to the different degrees of wettability on both sides of the membrane.^4^ Phospholipid membranes with symmetric structures exist extensively in cells and play an important role in various biological processes.^2^ However, asymmetric phospholipid microstructures, either membranes or tubes, could lead to novel properties and potential applications in biological processes. Hence, exploring asymmetric phospholipid microstructures with distinct two surfaces is worthwhile.

Phospholipid molecules have amphipathic properties with one hydrophilic functional group and one or two hydrophobic fatty acid groups in water solutions; these properties can be induced by solution molecules to form rich varieties of structures, such as membranes, tubes, and vesicles.^6–10^ For example, a series of lamellar structures, namely, membranes (including liquid crystallized, gel, and fluid lamellae), have been observed for phospholipids in water solutions.^6^ Phase diagrams of these structures have been also constructed using various parameters, where stable structures are separated by phase boundaries distinguished from first-order phase transitions.^8,11–14^ Previous experiments have indicated that water concentrations can induce the inverted hexagonal phase transiting to the lamellar phase by using complementary techniques of X-ray scattering and terahertz spectroscopy.^15^ Experimental results showed that the phase regions of the lipid membrane are determined by the water concentration in the water solutions.^8^ Other methods, such as electric field and shear flow, have also been used to expand the lamellar phase space in phase diagrams due to the special interest in lamellar structures. For example, under shear flows, lamellar microstructures can orient along the direction of the flows and enlarge the phase spaces for phospholipid membranes in the phase diagrams.^16^ These phase diagrams provide information on membrane structure distributions in the phase parameter space under various conditions at equilibrium states.

However, rapid processing usually causes polymers to reach non-equilibrium conformation, which leads to various polymer properties at different molecular levels; thus, the dynamics process plays an important role in deciding the material properties.^17^ Many recent experiments and computer simulations have revealed the dynamic processes of phospholipid membranes in solutions.^16–20^ For instance, increasing the content of branched fatty acids can increase the fluidity of membranes and change the kinetics of microbial membranes.^18^ Another research direction is to explore the mechanical properties, such as local pressure and interfacial permeability, of symmetric phospholipid membranes *via* experimentations and simulations.^21–25^ A previous experiment revealed the pressure response of cholesterol to solid-loaded phospholipid multilayers; it proved that cholesterol at a certain concentration decreases the tendency of separation and presents a new path for the construction of stable model membranes.^21^ Meanwhile, a theoretical approach based on self-consistent field theory has been developed to obtain the local stress across the membranes and interfaces in soft matter; this approach predicts the membrane lateral stress profile in the regions of the head and tail and the stress between hydrophobic and hydrophilic interfaces.^24^ Investigating asymmetric structures, either asymmetric particles or membranes, is meaningful. Many experiments and simulations have focused on asymmetric particles.^26–29^ Asymmetric particles have various potential applications in the biological field. For example, cancer therapeutic materials can be developed by studying Janus particles.^30^ The research scope should be extended to asymmetric membranes, tubes and vesicles, which exist naturally in biological systems, due to their asymmetric properties.

Several experimental and simulation studies have focused on asymmetric lipid membranes.^31–38^ For example, a plasma membrane with asymmetric complex structures was investigated in a previous work through large-scale molecular simulation (MD) simulations, where the membrane model included many lipid species that were asymmetrically distributed across the two leaflets.^35^ Another atomistic MD simulation revealed that short chains of one bilayer leaflet cannot interdigitate with those of the other leaflet and lead to increased free space in the middle of the bilayer.^36^ A coarse-grained (CG) MD simulation revealed that asymmetric structures assemble for multiple types of phospholipids in water solutions.^34^ These contributions concentrated on the structural complexity of individual membranes with multiple types of phospholipid molecules at either the atomistic or CG level. The kinetics and mechanical properties of asymmetric phospholipid membranes, which are relevant to practical applications, still need to be investigated.^39–41^ A recent experiment showed that the resulting microstructure can be altered by changing the chain length of phospholipid molecules.^42^ This finding suggests that the novel phenomenon probably appears in the self-assembly of multiple types of phospholipid molecules. In this work, we used the dissipative particle dynamic (DPD) method based on the CG model to predict the structural formation mechanism and performances of several asymmetric phospholipid microstructures in water solutions, which differs from the previous studies that examined the complexity of individual membranes. In the current work, we determined the asymmetric membranes, tubes and vesicles for the phospholipid polymers in the phase diagrams in terms of different chain lengths; we focused on the dynamic processes for the asymmetric phospholipid microstructures; we predicted the mechanical properties of asymmetric phospholipid membranes. Section II describes the model and method, and Section III presents the results and discussion. The summary is presented in Section IV.

## Method and model

### Method

The DPD method based on the CG model is suitable for simulating the hydrodynamic behavior of complex systems and soft matter.^11,43–46^ In DPD simulations, each DPD bead represents a cluster of molecules and obeys Newton's law. Forces, including conservation, dissipative, and random, appear in a pairwise manner between the *i*-th and *j*-th pairs of particles. Specifically, conservation force **F**^C^_*ij*_ is derived from a potential, dissipative force **F**^D^_*ij*_ is introduced to decrease the radial velocity difference between particles, and random force **F**^R^_*ij*_ is a stochastic force along the line connecting the particle centers. The total force on the *i*-th bead in the DPD system can be expressed as1
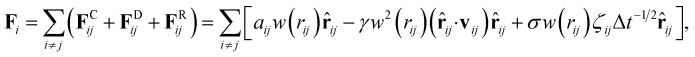
where the subscript index *ij* refers to the relative quantity between the *i*-th and *j*-th beads. *a*_*ij*_ is a particle interaction constant representing the maximum repulsive force between the *i*-th and *j*-th beads. *r*_*ij*_ is the relative distance, **r**_*ij*_ = **r**_*i*_ − **r**_*j*_ is the relative position, and **v**_*ij*_ = **v**_*i*_ − **v**_*j*_ is the relative velocity, where 
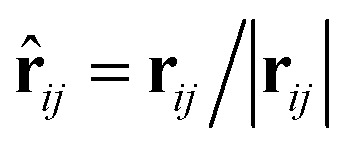
 between the *i*-th and *j*-th beads. *γ* is the friction coefficient governing the dissipative force, and *σ* is the noise amplitude determining the strength of random forces. The relationship between the two parameters is *σ*^2^ = 2*γk*_B_*T*, where *k*_B_ is the Boltzmann constant and *T* is the absolute temperature. *ζ*_*ij*_ represents a random uniformly distributed value between 0 and 1 with a Gaussian distribution and unit variance. Usually, standard values of *σ* = 3.0 and *γ* = 4.5 are used in simulations.^47^ The weight function *w*(*r*_*ij*_) can be expressed as2
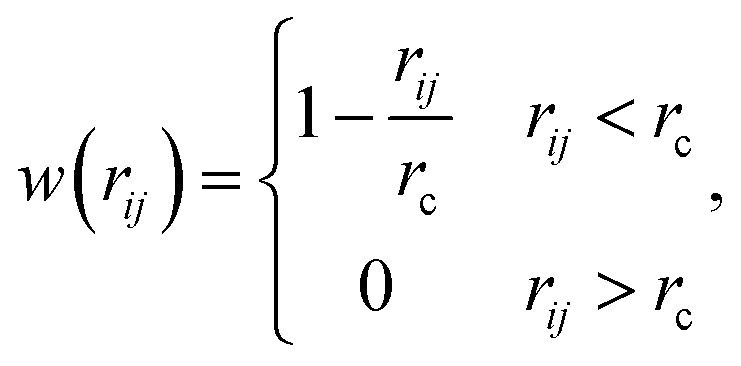
where *r*_c_ is a cut-off distance.

### Model

**Fig. 1:**
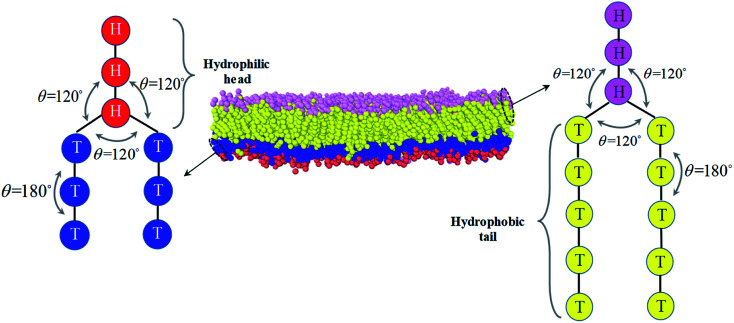
The schematic diagram for the phospholipid molecules with head group and two tail groups, as well as the asymmetric structure. The first type of phospholipid is demonstrated in the left, where the reds denote the head beads and blues are the tail beads. The second type of phospholipid is in the right, where the pinks represent the head beads and the yellows are the tail beads. The asymmetric membrane is taken as an example for the asymmetric structure and modeled by these types of phospholipid molecules is shown in the center.

An asymmetric membrane (Fig. 1) was developed based on the CG model by taking several carbon atoms together and grouping them into one bead. Two types of phospholipids (type one on the left and type two on the right in Fig. 1) were considered. The hydrophilic beads (HB) in types one and two are shown in red and pink, respectively, and the hydrophobic tails (TB) in types one and two are shown in blue and yellow, respectively. The two types of phospholipids are set to have one linear head chain and two linear tail chains, which have been extensively used in previous simulations.^15,16,48,49^ The bonded beads in phospholipids are connected by an additional elastic harmonic force3
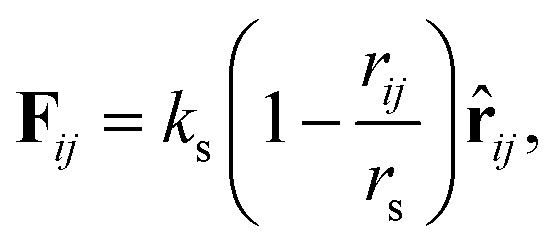
where *k*_s_ and *r*_s_ are the spring constant and equilibrium bond length between the *i*-th and *j*-th beads, respectively. In the present simulation, we used *k*_s_ = 100.0 and *r*_s_ = 0.7*r*_c_, which are similar to the values in previous studies.^48,49^ To achieve the bending force of the phospholipid molecule imposed on two consecutive bonds, we considered the following relationship:

4 **F**^*θ*^ = −*∇*[*k*_*θ*_(*θ* − *θ*_0_)^2^],

where *k*_*θ*_ is the bending constant and *θ*_0_ is the equilibrium angle. In the present simulation, we used *k*_*θ*_ = 6.0 and *θ*_0_ = π for three consecutive HBs or TBs in each chain, *k*_*θ*_ = 3.0 and 
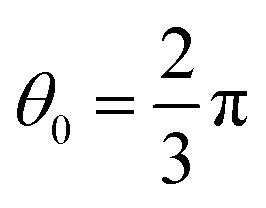
 between the head and tail chains are set for the last two beads in head chain and the first bead in tail chain, and *k*_*θ*_ = 4.5 and 
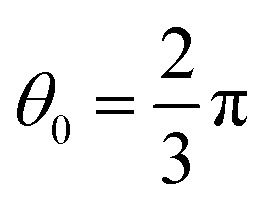
 between the two tail chains are set for the last one bead in head chain and the two first beads in each tail chain. We draws the schematic diagram for the phospholipid CG model in two-dimensional plane, as shown in Fig. 1. This model with multiple types of phospholipid membrane is similar to the symmetric membrane models used in previous studies.^50^

### Parameters

The simulations were performed in a cubic box with volume *V* = *L* × *L* × *L* under periodic boundary conditions. For simplification, we assumed the phospholipid and solvent water beads to have the same mass, and set this mass as the mass scale. We set the interaction cut-off radius for the phospholipid molecule and solvent beads *r*_c_ as the length scale, which is suitable for the phospholipid model. The cut-off radius can be estimated according to the expresses, *r*_c_ = (*ρV*_b_)^1/3^, where *ρ* is bead density and *V*_b_ is the volume of one DPD bead. We set *ρ* = 3 in our simulation, similar to previous simulations.^51^ The unit of time *τ* is defined as5
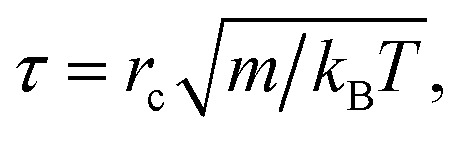
where *k*_B_*T* is the energy scale. In the current simulations, we set one DPD time step, Δ*t* = 0.01*τ*.

**Fig. 2:**
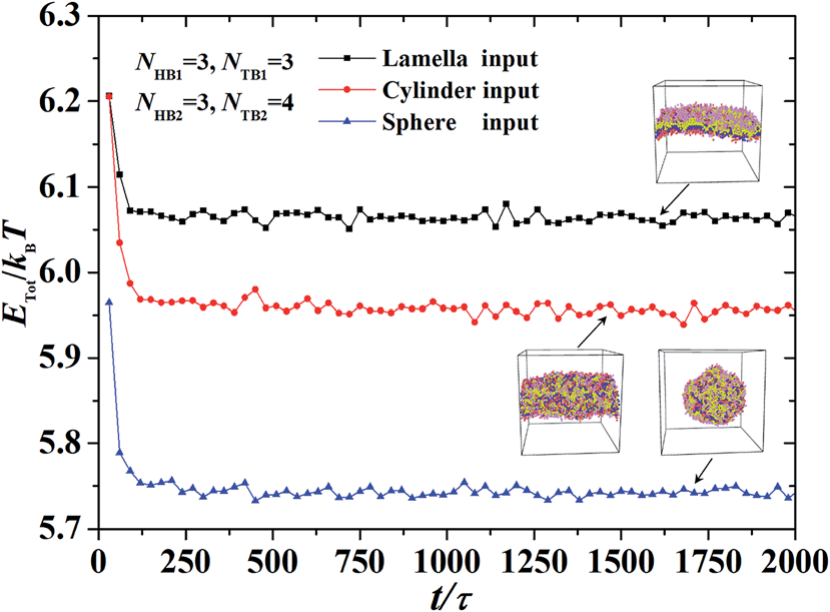
An example for obtaining the equilibrium and stable state in the dynamic processes, where the parameters are set to be *N*_HB1_ = 3, *N*_TB1_ = 3, *N*_HB2_ = 3, and *N*_TB2_ = 4. Three initial inputting distributions, *i.e.*, the lamella, cylinder and sphere inputting, are considered, respectively.

We took the repulsive interaction parameters *a*_*ii*_ = 25 for the same type of beads and *a*_*ii*_ = 100 for different types of beads; this value has been used extensively in previous simulations.^11,48,52^ Then, the Flory–Huggins parameter *χ*, was estimated by the relationship between *a*_*ii*_ and *a*_*ij*_, *i.e.*,*χ* = 0.286(*a*_*ij*_ − *a*_*ii*_), which has been adopted in previous simulations.^53–55^ To avoid the finite-size effect, we optimized the simulation box sizes by varying the box size *L* from 25*r*_c_ to 35*r*_c_. We selected the one with the lowest energy as the most optimized box size (please see an example in Fig. S1 and S2 of the ESI†). All simulations were performed on NVT ensembles, and the numbers of chains for the first and second types of phospholipids were set to *n*_1_ = *n*_2_ = 600. Usually, the system can reach the equilibrium state when the process is about 200 000 DPD time steps, as shown in Fig. 2. At this state, the energy decreases to the lowest value. Moreover, pressure and temperature were uniformly distributed in the simulation boxes, similar to a previous simulation.^16^ An exmaple data is shown in Fig. S3 of the ESI,† where the pressure and temperature distribute uniformly in the space. For the same system with fixed parameters, we inputted different initial distributions of phospholipid chains, which led to different output energies in the system. Then, we selected the one with the lowest energy as the final stable structure for this parameter point. For example, we inputted three initial chain distributions, *i.e.*, lamellar, cylindrical, and spherical, and obtained three output energies in one phase parameter point, as shown in Fig. 2. After comparing the output energies, we selected the one with the lowest energy as the stable structure.

## Results and discussions

**Fig. 3:**
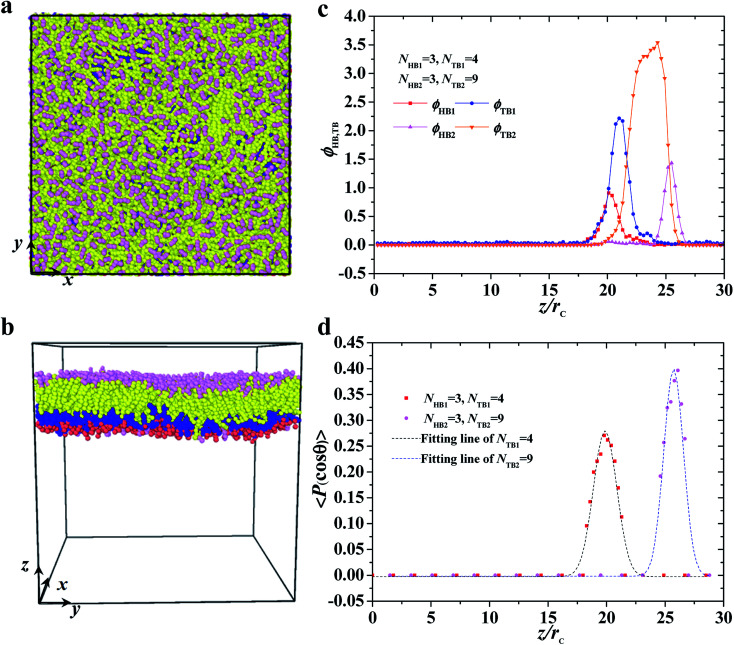
Typical asymmetric membrane with *N*_HB1_ = 3, *N*_TB1_ = 4, *N*_HB2_ = 3, and *N*_TB2_ = 9. Snapshot for the phospholipid bilayer with (a) the top view and (b) side view are demonstrated, and (c) the density profiles of head and tail beads and (d) the order parameters are shown along the *z* axes. The dotted line indicates the fitting line.

**Fig. 4:**
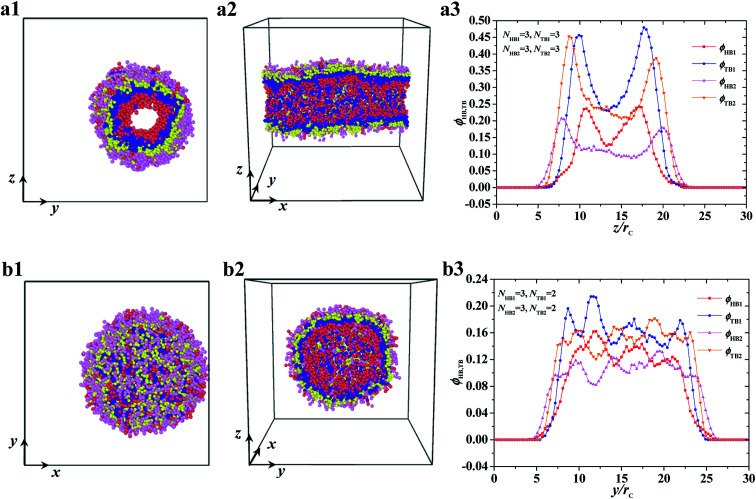
Typical asymmetric tube and vesicle. (a1–a3) tube with *N*_HB1_ = 3, *N*_TB1_ = 3, *N*_HB2_ = 3, and *N*_TB2_ = 3, and (b1–b3) vesicle with *N*_HB1_ = 3, *N*_TB1_ = 2, *N*_HB2_ = 3, and *N*_TB2_ = 2. The top view and side view are list while the density profiles of head and tail beads are list in the right.

**Fig. 5:**
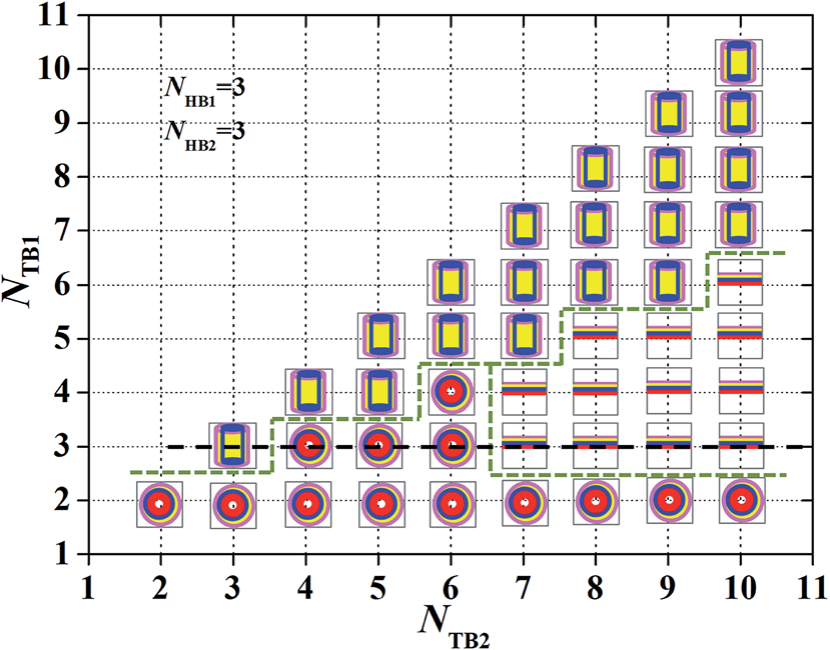
Characteristic for the asymmetric structures depending on the chain length. A phase diagram with *N*_HB1_ = 3 and *N*_HB2_ = 3, in terms of the tail chain length, *N*_TB1_and *N*_TB2_. The symbols 
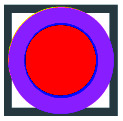
, 
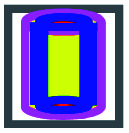
, 
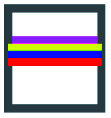
 represent the asymmetric vesicle, asymmetric tube and asymmetric membrane, respectively.

**Fig. 6:**
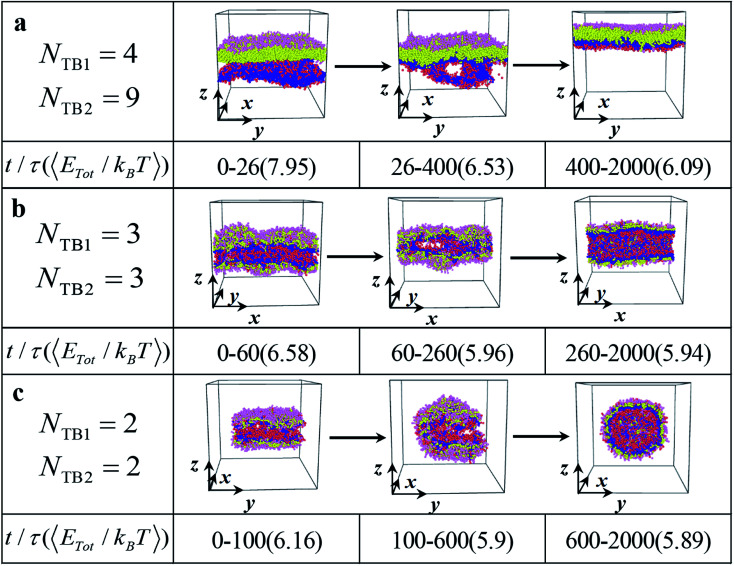
The dynamic processes for the typical (a) asymmetric membrane with *N*_TB1_ = 4 and *N*_TB2_ = 9, (b) asymmetric tube with *N*_TB1_ = 3 and *N*_TB2_ = 3, and (c) asymmetric vesicle *N*_TB1_ = 2 and *N*_TB2_ = 2. The typical structures are shown in three stages with the average energies and duration times list below.

**Fig. 7:**
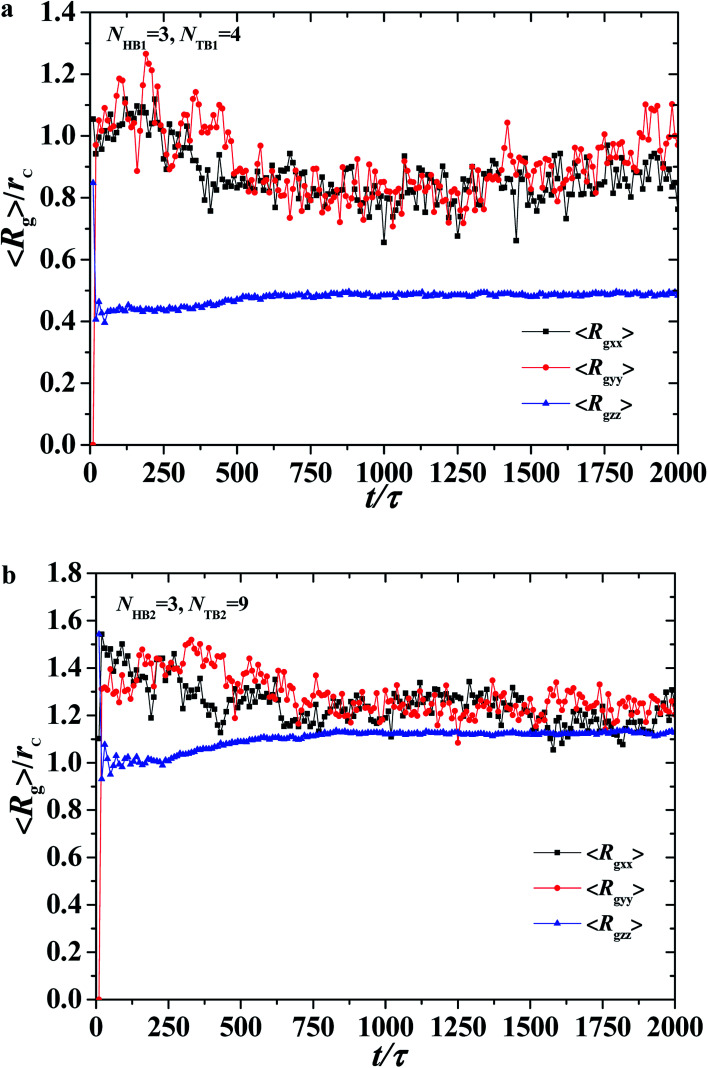
The average radius of gyration of the asymmetric membrane with *N*_HB1_ = 3, *N*_TB1_ = 4, *N*_HB2_ = 3, and *N*_TB2_ = 9. Three components for (a) the first type of phospholipid chain and (b) the second type of phospholipid chain as functions of time steps.

**Fig. 8:**
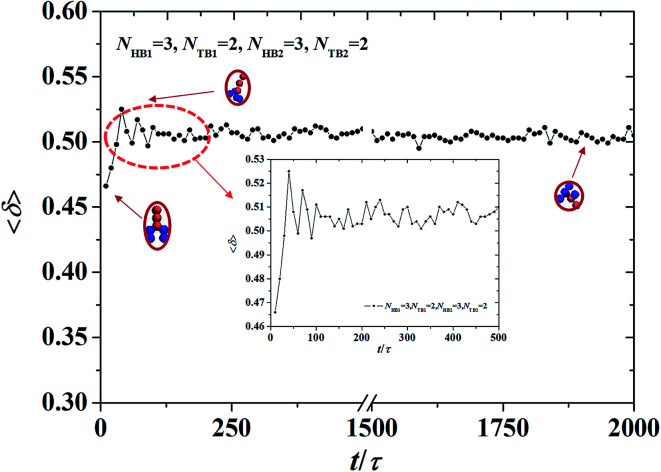
The shape factor of the asymmetric vesicle with *N*_HB1_ = 3, *N*_TB1_ = 2, *N*_HB2_ = 3, and *N*_TB2_ = 2. The data circled by the red dotted box is enlarged and shown in the inserted.

**Fig. 9:**
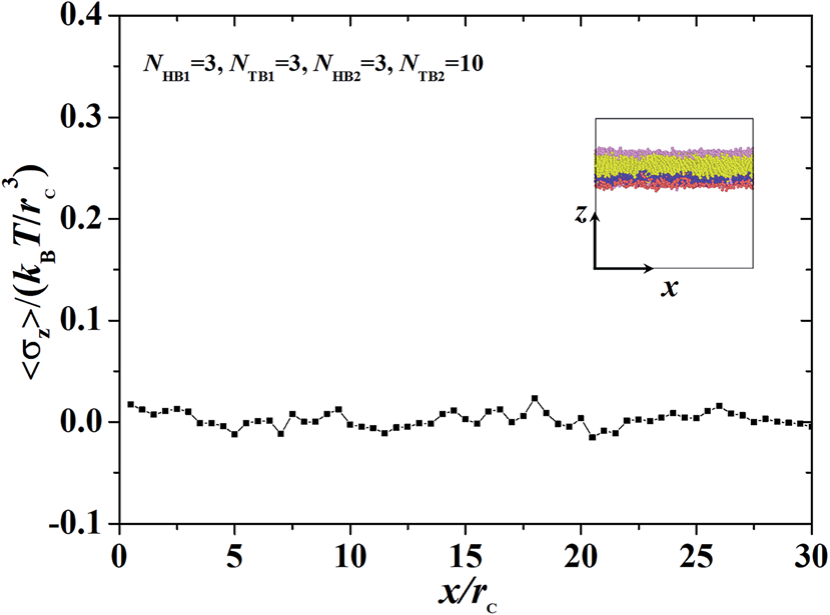
The interface tension 〈*σ*_*z*_〉 as function of distance along *x*-direction for the asymmetric membrane, with *N*_HB1_ = 3, *N*_TB1_ = 3, *N*_HB2_ = 3, and *N*_TB2_ = 10. The asymmetric membranes are also inserted.

**Fig. 10:**
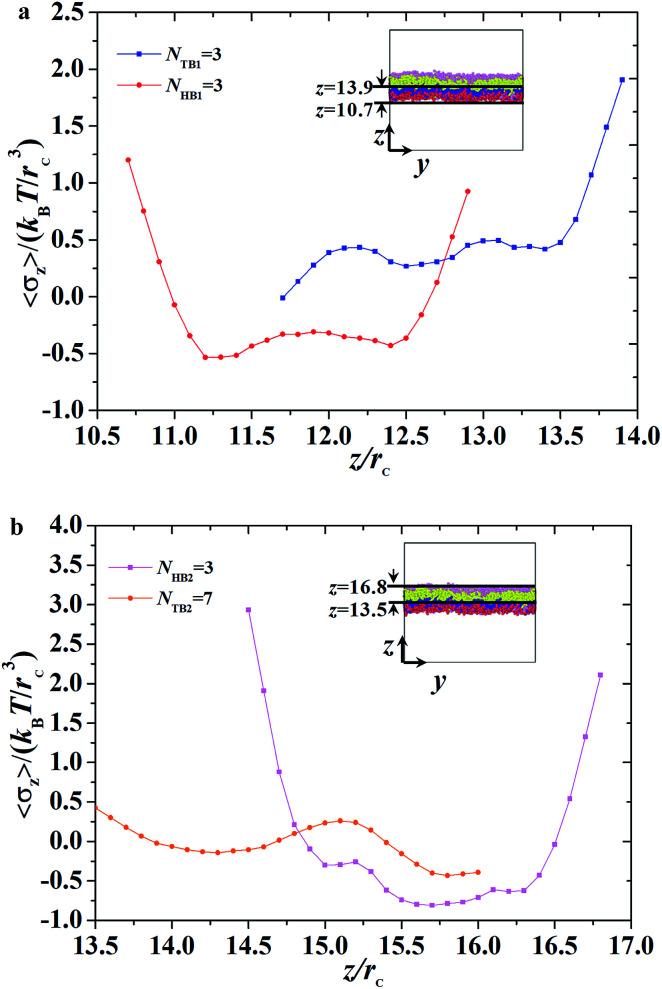
The interface tension 〈*σ*_*z*_〉 as function of distance along *z*-direction for the asymmetric membrane, with *N*_HB1_ = 3, *N*_TB1_ = 3, *N*_HB2_ = 3, and *N*_TB2_ = 7. (a) The first type of phospholipid chain, (b) the second type of phospholipid chain. The asymmetric membranes are also inserted.

**Fig. 11:**
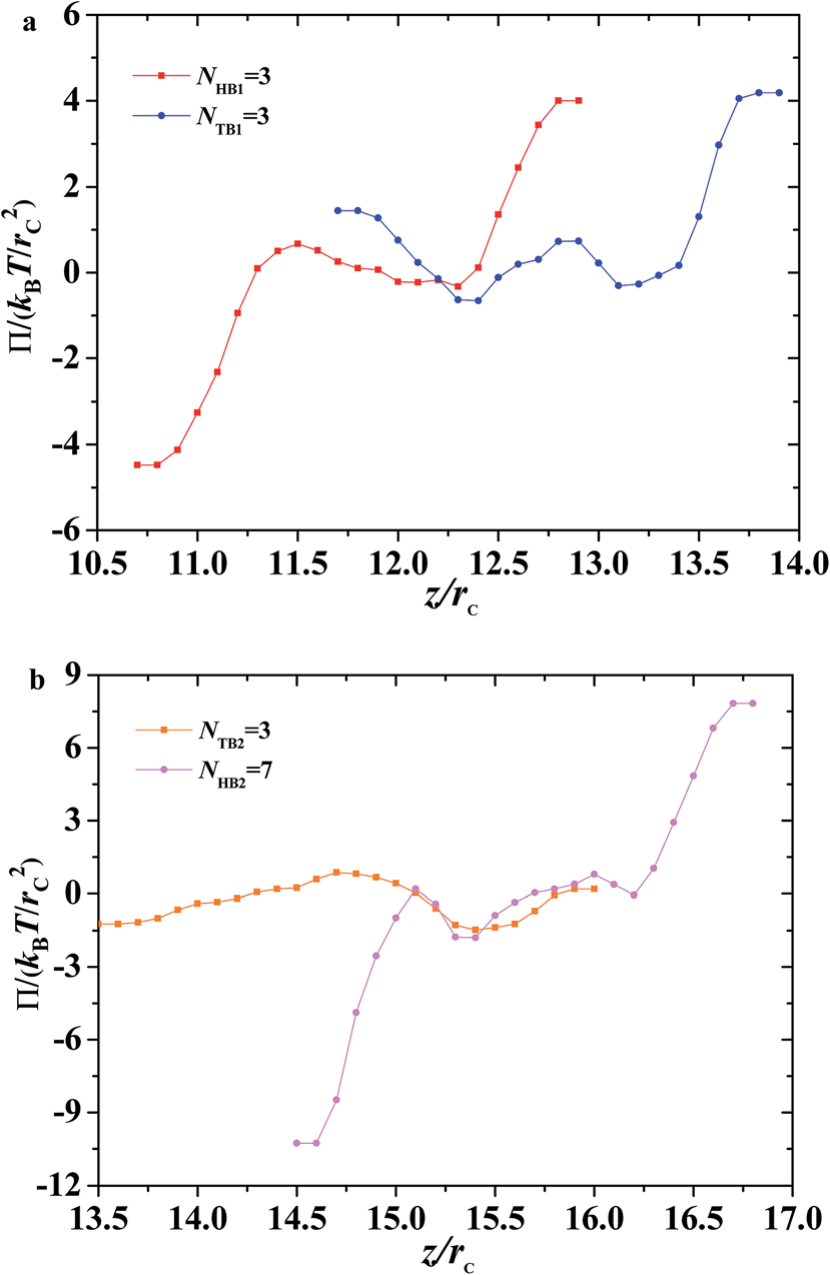
Osmotic pressure as function of distance along *z*-direction for the asymmetric membrane with *N*_HB1_ = 3, *N*_TB1_ = 3, *N*_HB2_ = 3, and *N*_TB2_ = 7. (a) The first type of phospholipid chain, (b) the second type of phospholipid chain.

For a system with two types of phospholipid chains in water solutions, the parameter space is too large to explore. Hence, we reduced the globular space into a simplified parameter space by fixing several parameters. In particular, we fixed the interaction parameter (*a*_*ij*_), numbers of phospholipid chains (*n*_1_ and *n*_2_), and head bead numbers (*N*_HB1_ = *N*_HB2_ = 3). Meanwhile, we varied the tail bead numbers for the two types of phospholipid chains (*N*_TB1_ and *N*_TB2_) from 2 to 10. We concentrated on the observed asymmetric structures (Fig. 3–5). The corresponding dynamic processes (Fig. 6–8) and mechanical properties (Fig. 9–11) were derived by varying the tail bead numbers.

### Asymmetric structures and phase diagrams

This subsection discusses three types of asymmetric structures, *i.e.*, asymmetric membrane, asymmetric tube, and asymmetric vesicle. Fig. 3 shows a typical asymmetrical membrane structure with parameters of *N*_TB1_ = 4 and *N*_TB2_ = 9. This asymmetric bilayer membrane constitutes two types of monolayer membranes (top and side views shown in Fig. 3a and b, respectively). Naturally, the hydrophilic head beads are arranged near the outer water regions, and the hydrophobic tail beads are packed into the center regions inside the membranes due to the amphiphilicity of phospholipid molecules in water solutions. To intuitively describe the asymmetric membrane structure, we plotted the density distributions of the hydrophilic and hydrophobic beads along the *z*-direction, as shown in Fig. 3c. The density profiles for the head and tail bead distributions have four peaks. The peaks in the head bead profiles are separated for the first and second types of phospholipids, which clearly demonstrates the bilayer structures in the membranes. Meanwhile, the peaks overlap in the tail bead profiles, which indicates that the tail chains are interdigitated for the two types of phospholipids. Such a bimodal structure has also been confirmed in previous phospholipid membrane simulations.^56–58^ In addition, we investigated the orientation ordering of the head beads for asymmetric phospholipid membranes as follows:^59,60^6
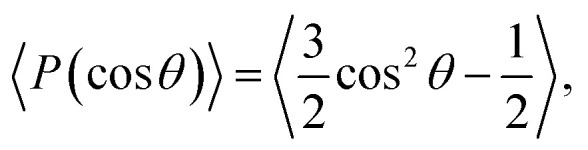
where *θ* is the angle between the chain direction and the *z*-axis direction and the bracket represents an ensemble average. In general, when the direction of the chain is completely parallel to the *z*-axis, the value of the degree of order is equal to 1. When the direction of the chain is completely perpendicular to the *z*-axis, the value of the degree of order approaches −0.5.^11^ The orientation distributions are shown in Fig. 3d, where the red dot represents the simulation results of the first type of head beads, the pink dot denotes the simulation results of the second type of head beads, and the dotted lines are the fitting results. The fitting curve is close to the Gaussian distribution, and the simulation value is between 0 and 0.5, but not 0. These facts indicate that the angles between the chain length and *z*-axis are between 0 and π, thus showing that the asymmetrical phospholipid membranes have well-ordered structures. However, the order degree of head bead is bigger for the second type of phospholipid than those of the first type phospholipid. Although the numbers of head beads are the same for these two type of phospholipids, they have different tail chains. The longer tail chains for the second type phospholipid, *i.e.*, *N*_TB2_ = 9, have more obvious effects on the ordering degree of the head chain than those with the short tail chain of *N*_TB1_ = 4.

The two other asymmetric structures, namely, asymmetric tube and asymmetric vesicle, are shown in Fig. 4. The top and side views and bead density distributions of the two typical asymmetric structures are presented. The side and spatial cross-sectional views of the asymmetric tube structure, with parameters of *N*_TB1_ = 3 and *N*_TB2_ = 3, are shown in Fig. 4a1 and 4a2, respectively. From these cross sections, we observed that the cylindrical tube has an asymmetric structure, with the first type of beads distributed in the inner layer. To analyze the spatial structure of the asymmetric tube, we plotted the density profile of the beads along the *z*-axis cross-section in Fig. 4a3. For the inner layer, the double peaks appear in the profiles of head and tail bead distributions, *i.e.*, *ϕ*_HB1_ and *ϕ*_TB1_, which indicates that the asymmetric column is hollow. The peak of tail bead profile *ϕ*_TB1_ for the inner layer is close to the peak of the tail bead profile in outer layer *ϕ*_TB2_. This fact indicates that the chains in the two layers overlap. For the asymmetric vesicle, we plotted front and spatial cross-sectional views, as shown in Fig. 4b1 and 4b2, respectively. Similarly, the spherical vesicle has a bilayer structure, with the first type of chains in the inner regions and the second type of chains in the outer regions forming a hollow spherical-like structure. This distribution is due to the hydrophilic heads and hydrophobic tails of the phospholipid molecules in water solutions. Then, we analyzed the asymmetric spherical structure by plotting the bead density distributions in the *y*-axis cross-sectional direction, as shown in Fig. 4b3. Although the double peaks in these bead profiles are not obvious, we could still distinguish the bilayer structures from the obvious peaks near the outer regions, and the phospholipid molecules are distributed in a certain order. Our observations on the asymmetric vesicles and membranes are similar to those observed for planar and vesicle membranes in multiple types of phospholipids, among which POPC, POPE, POPS, PPCS, CHOL, and fatty acid molecules with fixed chain lengths participate in assembly.^34^ However, we observed the cylindrical membranes, *i.e.*, the tubes, for only two types of phospholipids in water solutions. We focused on the structure distributions that depended on chain lengths for the two types of phospholipids.

To observe such a chain length effect, we changed the tail chains for the two types of phospholipids and constructed the phase diagrams, as shown in Fig. 5. Here, we assume only three candidate phases, the asymmetric membrane, asymmetric tube and asymmetric vesicle, appearing in the phase diagram. In particular, we changed the number of tail beads in the simulation to range from *N*_TB1_ = 2–10 and *N*_TB2_ = 2–10. Given that the number of beads in the two tail chains is symmetrical, only half of the phase diagrams are plotted. In the phase diagrams, three phase regions are divided with a dotted line for visual guidance, and the asymmetric membranes, asymmetric tubes, and asymmetric vesicles are separated. The phase distributions in the phase diagram have several characteristics. The first one is that the asymmetric vesicles are located in the bottom of the phase diagram where the tail chains are shorter for one type of phospholipid molecules, mostly even with *N*_TB1_ = 1, as shown in Fig. 5. Usually, spherical structures have high curvatures, which need high asymmetric components in the inner and outer regions. This condition requires small numbers of tail beads in the outer regions and large numbers of tail beads in the inner regions. The second characteristic is that the asymmetric tube is distributed near the diagonal regions where the tail chains are nearly equal to the two types of phospholipids. This distribution indicates that the two types of tail chains have similar features and are inputted into the center regions, whereas the head chains are attracted to the outermost and innermost regions by the water molecules. The third characteristic is that the asymmetric membranes appear in regions where one tail chain is long, whereas the other chains are short. This condition indicates that the relatively long tail chains can easily form such structures. This fact guides us in obtaining asymmetric membranes for multiple types of phospholipid molecules in water solutions. In additional, we have studied the change in the average radius of gyration of the structure with *N*_TB1_ = 3 along the dotted line in Fig. 5 (see Fig. S4 in the ESI†). Here, the average radius of gyration will be defined in eqn (7) and (8) in the next subsection. The results indicated that the average radius of gyration initially decreases as *N*_TB2_ increases, where the structure changes from an asymmetric tube to an asymmetric vesicle. Then, the average radius of gyration smoothly increases as *N*_TB2_ increases when the structures are always asymmetric membranes.

### Dynamic process

We investigated several typical dynamic processes of asymmetric structures, namely, asymmetric membrane, asymmetric tube, and asymmetric vesicle, with a time step. Three examples are shown in Fig. 6, where the main stages are listed with evolutionary states and time and the corresponding average energies. First, all three dynamic processes of asymmetric structures can be divided into three stages: random generation, mutual adaptation, and formation. Specifically, for asymmetric membrane the initial random stage lasts for 26*τ* at a relatively high energy of 7.95*k*_B_*T* and subsequently evolves into the mutual adaption stage, which sustains a relatively long time of 374*τ* at a low average energy of 6.53*k*_B_*T*, as shown in Fig. 6a. In this stage, the structure is continuously adjusted and evolved. Afterward, the system develops into the structure-formatting stage, where the relatively regular membranes are completely formatted with the lowest energy of 6.09*k*_B_*T*. The energy is gradually reduced and tends to stabilize during the formation of the asymmetric membrane, which indicates that the asymmetric membrane structure in the formation stage has reached a stable structure. This evolution process is similar to that in previous simulations.^11,56^ In the dynamic processes for the asymmetric tube and vesicle, as shown in Fig. 6b and c, the durations and energies differ from those observed in the asymmetric membranes. The energy changes in the three stages are 6.58*k*_B_*T*, 5.96*k*_B_*T*, and 5.94*k*_B_*T*, which tend to stabilize the structure as the time step increases, as shown in Fig. 6b. The random generation phase of the asymmetric tube structure is longer than that of the asymmetric membrane structure, but the asymmetric tube structure can enter the formation phase more quickly. This result suggests that the process can rapidly drown in the polymer fiber where the polymer chains are highly stretched.^17^ Our observation on the asymmetric tube agrees with this prediction. For the dynamic process of the asymmetric vesicle structure, as shown in Fig. 6c, the structure occludes the upper and lower parts, gradually joins the connection over time, and finally forms a hollow asymmetric spherical structure with energy changes of 6.16*k*_B_*T*, 5.90*k*_B_*T*, and 5.89*k*_B_*T*. The asymmetric spherical structure requires a longer simulation time to achieve a stable structure compared with the asymmetric membrane and asymmetric cylinder.

To describe the dynamic process in detail, we considered the average radius of gyration 〈*R*_g_〉. The radius of gyration tensor **R**^2^_g_ can be expressed as^61^7
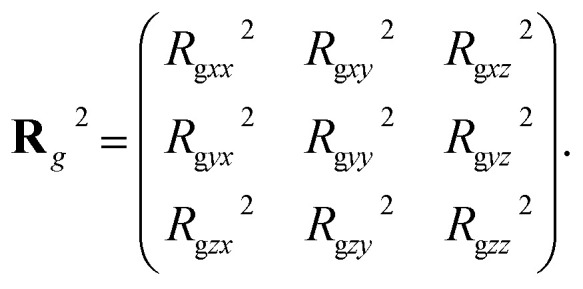


The elements *R*^2^_g*αβ*_ can be written as8
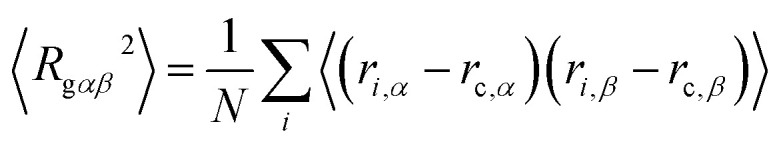
with *α*,*β*∈{*x*,*y*,*z*};*N* and *r*_*i*,*x*_ are the number of chains and *x*-coordinate of *i*-th bead, respectively; and *r*_c_ is the mass center. In the current simulations, we focused on the average radius of gyration of the asymmetric membrane, as shown in Fig. 7, where the parameters are *N*_TB1_ = 4 and *N*_TB2_ = 9. The average radius of gyration for the two types of phospholipid molecules are shown in Fig. 7a and b. The average value of 〈*R*_g*zz*_〉 is smaller than those of 〈*R*_g*xx*_〉 and 〈*R*_g*yy*_〉, and the average values of 〈*R*_g*xx*_〉 and 〈*R*_g*yy*_〉 are basically the same, indicating that the polymer chains are compact in the *z*-direction and stretched in the *x*- and *y*-directions, as shown in Fig. 7a. During the self-assembly period, the average values of 〈*R*_g*xx*_〉 and 〈*R*_g*yy*_〉 increase initially then decrease to stable values, as shown in Fig. 6a. This result is consistent with the microstructural dynamics observed in Fig. 6a, which is a layered distribution on the *x*–*y* plane. For the second type of phospholipids, which is unlike the first type, the average radius of gyration in the *z* direction 〈*R*_g*zz*_〉 is almost the same as those in the *x*- and *y*-directions, 〈*R*_g*xx*_〉 and 〈*R*_g*yy*_〉. The values of 〈*R*_g*xx*_〉 and 〈*R*_g*yy*_〉 in the *x*- and *y*-directions are basically the same, and they smoothly decrease to stable values in the structural formation stage. As observed in Fig. 6a, the second type of phospholipids is distributed in the above layers, most parts of which complete the chain arrangement in the last two stages. A small adjustment was observed in the distances between the chain beads, where the distance between the beads decreases and becomes compact. This result is similar to the formation process we observed previously in phospholipid symmetric membranes.^16^

Furthermore, we extracted the shape factor of the structure by using the average radius of gyration and investigated its evolution in the dynamic processes. Shape factor 〈*δ*〉 can be expressed as follows:^62,63^9
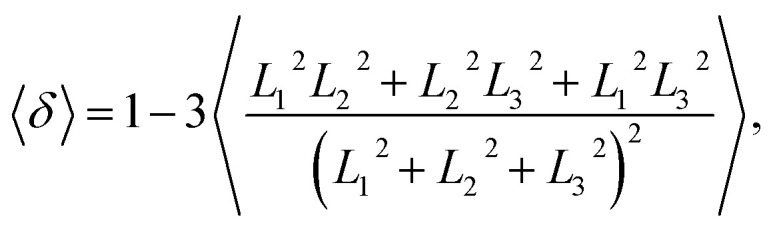
where *L*_1_^2^, *L*_2_^2^, and *L*_3_^2^ are the three eigenvalues of the square radius of gyration tensor **R**_g_^2^. For convenience, we sorted them as *L*_1_^2^ ≤ *L*_2_^2^ ≤ *L*_3_^2^. We investigated the shape factor for the chains in the asymmetric vesicle. An example is shown in Fig. 8, where the parameters are set to *N*_TB1_ = 2 and *N*_TB2_ = 2. The shape factor increases then stabilizes at 0.5, as shown in Fig. 8, where the chain conformations are also plotted. In the beginning, the chain exhibits an elliptical conformation then a circular conformation at the stable stage. This is evidence that shape factor 〈*δ*〉 begins at 0.47 and then approaches 0.5 at the stable stage, a result that is similar to previous simulation results.^64^ To observe the shape factor in detail, we drew the variation of the shape factor between 0*τ* and 500*τ* as the inserted part. By investigating the variation of the shape factor, we found that the asymmetric vesicle structure easily forms when the structure of the single chain exhibits spherical conformation.

### Mechanical properties

We focused on the mechanical properties of asymmetric membrane structures in solutions by analyzing the interface tension. The interface tension of the phospholipid symmetric membrane has elicited much interest in recent years.^25,65,66^ According to the Irving–Kirkwood definition, the formulation of tension *σ*_*z*_ along the *z* direction is as follows:^67^10
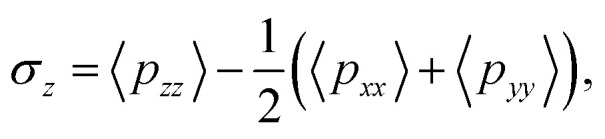
where the element *p*_*xx*_ is a component of the pressure tensor and can be achieved as11
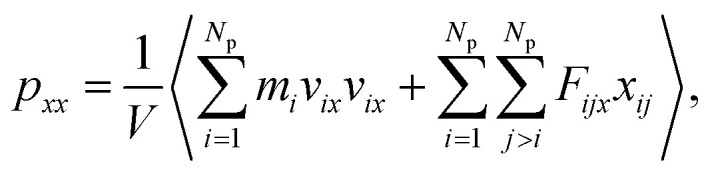
where *N*_p_ is the number of DPD particles and *F*_*ijx*_ and *x*_*ij*_ are the force and relative position between particles *i* and *j* along the *x*-direction. Elements *p*_*yy*_ and *p*_zz_ have the same definition, except for the replacement of the corresponding subscripts. Fig. 9 shows the surface tension along the *x*-direction of an asymmetric membrane structure with parameters *N*_TB1_ = 3 and *N*_TB2_ = 10, where surface tensions *σ*_*z*_ are averaged over the *y*- and *z*-directions. The average value of *σ*_*z*_ is nearly equal to zero, indicating that the asymmetric membrane is a free and planar membrane in the solutions despite of *x* changes. This result is similar to those of previous theoretical and simulation observations of symmetric membranes for phospholipids in solutions.^11,68^

Given that the interface tensions are equal to zero by taking the average over *y*- and *z*-directions, the detailed distribution in the *z*-direction is still necessary. We investigated the mechanical properties for the asymmetric membrane by calculating the interface tension distributed along the *z*-direction for the two types of phospholipid molecules, as shown in Fig. 10. The example parameters were set to *N*_HB1_ = 3 and *N*_TB1_ = 3 for the first type of phospholipids and to *N*_HB2_ = 3 and *N*_TB2_ = 7 for the second type. As shown in Fig. 10a, first-type phospholipid molecules are predominantly distributed in a range of 10.7–13.9*r*_c_, leading to the distribution of interface tension in the same region. For the head beads, the interface tension decreases in the beginning then increases, namely, the interface tension is distributed in the interfacial regions between the mixing molecules. However, the interface tension only exists near the regions between the water and phospholipid molecules for the tail beads. This condition means that the tensions in the interface regions between the head and tail chains are mainly contributed by the parts of the head chains because they are more rigid than those of the tail chains. The second type of phospholipids, which is mainly distributed in the regions from 13.5*r*_c_ to 16.8*r*_c_, makes up the other asymmetric layer, as shown in Fig. 10b. Similarly, we plotted the interface tensions at the tail and head chains for the second type of phospholipids, as shown in Fig. 10b. The head chains have similar distributions as those in the first type due to the rigidity of the head chains, but the tension of the tail chains only fluctuates within a small value. This result was obtained probably because the second type has more flexibility than the first type; the tail chains are longer for the second type than for the first type. Comparison of the interface tensions of the two types of phospholipids shows that the interior of the asymmetric membrane structure is essentially in a tension-free state, but interfacial tension exists near the boundary.

In addition to surface tension, the permeability of the asymmetric membrane structure is also important. The formula for calculating osmotic pressure is as follows:12
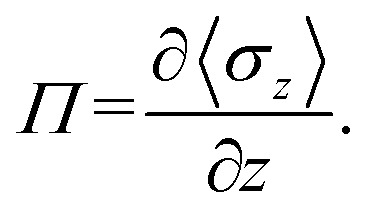


Here, we plotted the osmotic pressures as functions of position *z* for the asymmetric membrane, as shown in Fig. 11, where the parameters are set to *N*_TB1_ = 3 and *N*_TB2_ = 7, the same as those in Fig. 10. For the first type of phospholipids, the two peaks appear in the two interfacial regions between the head beads and water beads, head beads and tail beads, respectively, as shown in Fig. 11a. However, the tail beads mainly contribute one maximum value near the tail beads from the second type of phospholipid. Actually, the osmotic pressure results from the gradient distribution of interfacial tension, it obviously has the maximum value where the interfacial tension varies most intensively, according to the distributions in Fig. 10a. Similar cases were observed for the second type of phospholipids, as shown in Fig. 11b. The difference is that the osmotic pressure in the center regions results from the head chains not the tail chains. Namely, the tail beads of the second type phospholipid contribute a very small percentage for the osmotic pressure, which is probably due to its short length. This result might help us understand the non-directional transmission through asymmetric membranes.

## Summary

In this work, we used DPD simulation to predict an asymmetric membrane, asymmetric tube, and asymmetric vesicle for phospholipids in aqueous solutions. The asymmetric structures assembled from two types of phospholipid molecules were investigated by analyzing their bead density distributions and order parameters. The results showed double layers in these asymmetric structures and good ordering near the boundaries. The asymmetric structures distributed in the phase diagram were also analyzed by changing the tail chain lengths of the two types of phospholipids. The phase diagram indicated that the asymmetric tubes were distributed along the diagonal regions, and the asymmetric membranes were located in the regions with long tail chains due to the chain asymmetry and amphiphilicities in the solutions. However, the phase diagram in the current work is limited in the variances of tail chain lengths. We expect a richer phase behavior when the parameters of the system expand beyond those considered here. For example, we assumed comparable interactions between the beads, *i.e.*, the Flory–Huggins parameter. When the Flory–Huggins parameter become stronger, we expect that the membrane phase can be extended in the phase diagram. In particular, our observation on the subset of the full parameter space is a starting point to explore the phase behavior of asymmetric structures in the full parameter space.

Then, we investigated the dynamics of the asymmetric structures and observed the formation of the asymmetric membrane, asymmetric tube, and asymmetric vesicle in the solutions. The structural evolution was divided into three stages for the three asymmetric structures. The stages were initial random, adaptation, and formation, which were determined by the energy of the structure. Furthermore, the average radius of gyration of the asymmetric membrane structure was investigated. The dynamic formation process of the asymmetric membrane structure was analyzed, and the formation process of the membrane was evaluated intuitively. The shape factor of the asymmetric vesicle structure proved that the self-assembly of the conformation phospholipid molecules formed a spherical conformation.

We also investigated the mechanical properties of the asymmetric membrane in solutions by analyzing the interface tension and osmotic pressure. Evaluation of the mechanical properties of the asymmetric membrane structure revealed that the average tension for the membrane structure was zero, but interface tension and osmotic pressure were distributed along the *z*-direction. The simulation suggested that tension and pressure existed near the boundaries between the phospholipid and water domains due to the rigidities of the head chains. This work provides a method of designing asymmetric membranes in biological processes and understanding the mechanism of directional transport *via* membranes.

## Conflicts of interest

There are no conflicts to declare.

## Supplementary Material

RA-010-D0RA03732J-s001
