# Supplementary material for: Predicting asymmetric phospholipid microstructures in solutions
Source: RSC Adv. 2020 Jun 26;10(41):24521–32. doi: 10.1039/d0ra03732j (PMC9055179; doi:10.1039/d0ra03732j)
Supplement: RA-010-D0RA03732J-s001 [file RA-010-D0RA03732J-s001.pdf]

Electronic Supplementary Material (ESI) for RSC Advances.
This journal is © The Royal Society of Chemistry 2020

Predicting asymmetric phospholipid microstructures in solutions

Yue Shan[a], Yongyun Ji[a], Xianghong Wang[a], Linli He[a], and Shiben Li[*, a]

[a] *Department of Physics, Wenzhou University, Wenzhou, Zhejiang 325035, China.*

*Authors to whom correspondence should be addressed.

Electronic addresses: *shibenli@wzu.edu.cn* (S. Li)

Fig. S1

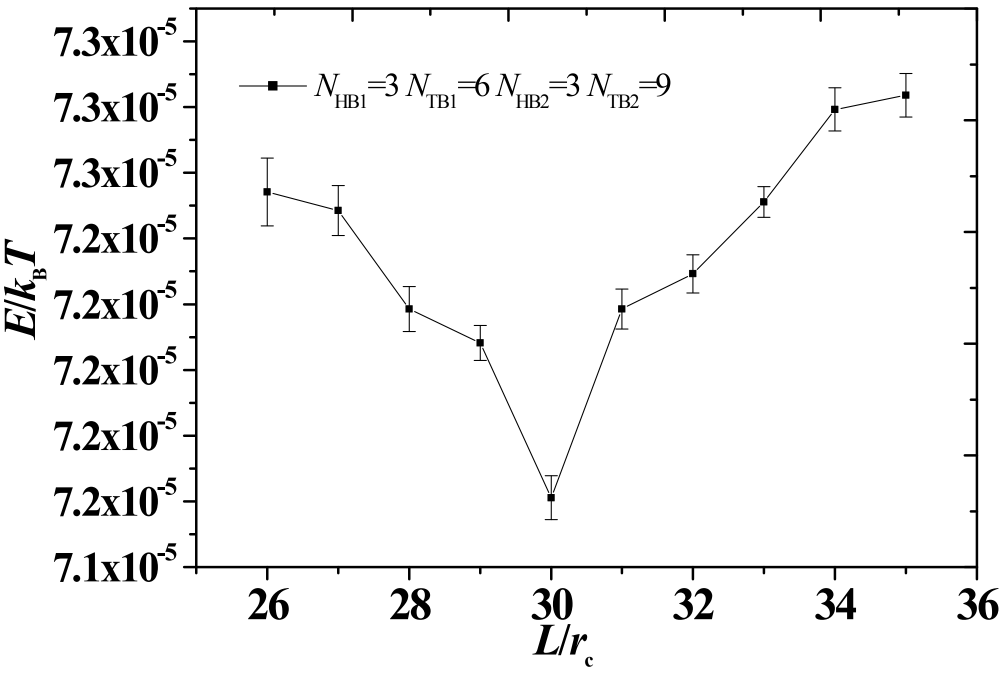

Fig.S1 The average energy of molecule as function of box size L. The phospholipid with the parameter of $N_{HB1}=3$, $N_{HB2}=3$, $N_{TB1}=6$, and $N_{TB2}=9$.

Fig.S2

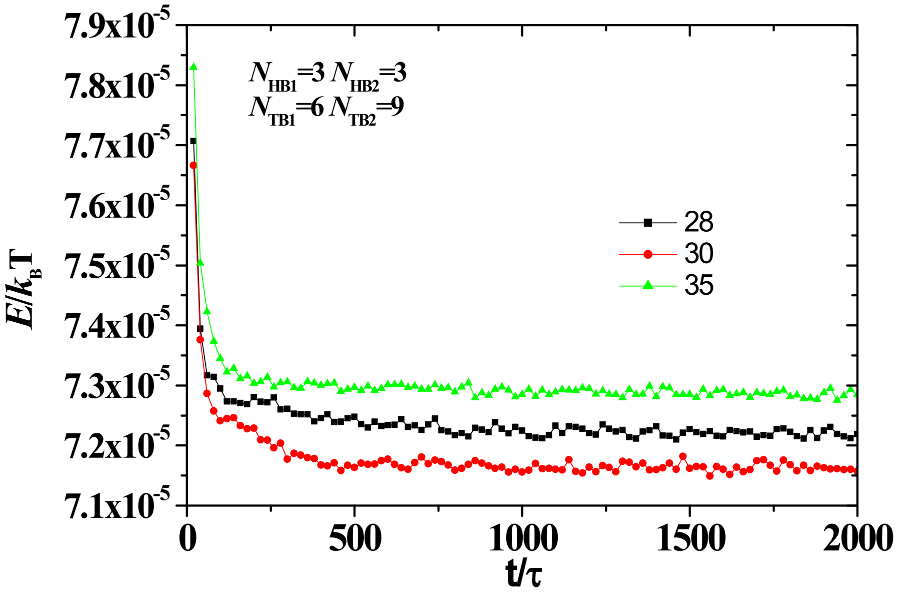

Fig.S2 The average energy of molecule as function of time step. The phospholipid with the parameter of $N_{HB1}=3$, $N_{HB2}=3$, $N_{TB1}=6$, and $N_{TB2}=9$. The box sizes L are set to be $L=28r_c$, $30r_c$ and $35r_c$.

Fig.S3

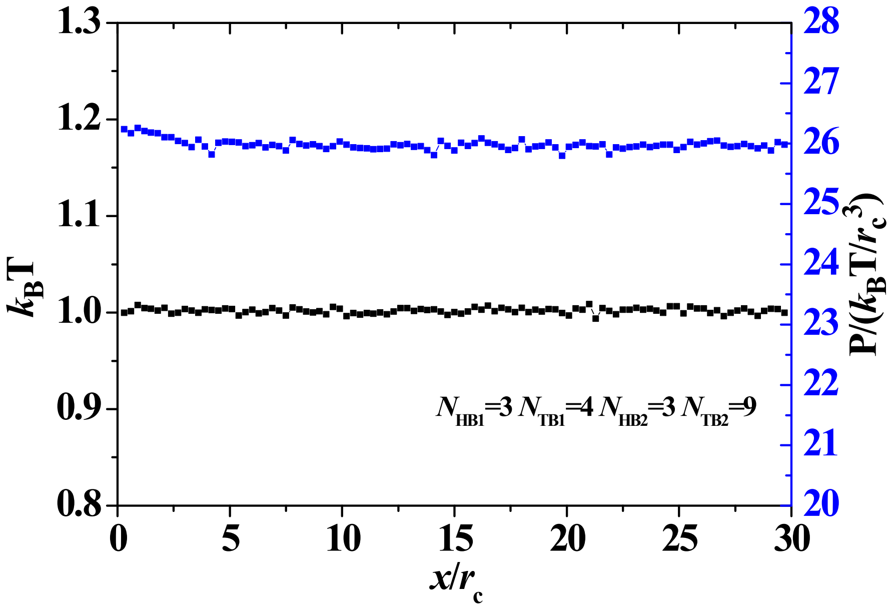

Fig.S3 The local pressure P and temperature T as functions of x.

Fig.S4

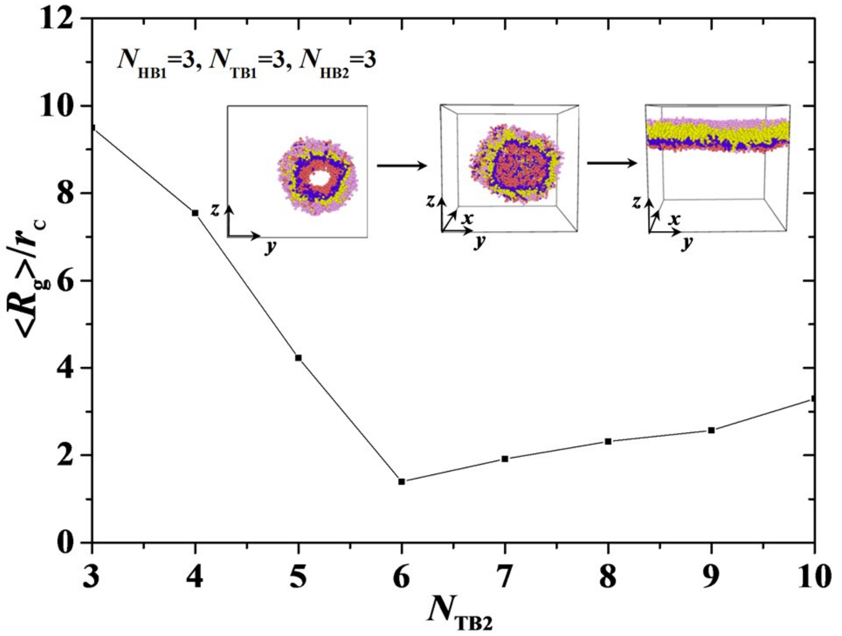

Fig.S4 The average of radius of gyration as a function of N_{TB2}, with $N_{HB1} = 3$, $N_{TB1} = 3$, and $N_{HB2} = 3$ The corresponding asymmetric structures are also inserted.
